# Pathological and Surgical Observations on Diseases of the Joints

**Published:** 1840-10

**Authors:** 


### Art. II.

1. *Pathological and Surgical Observations on Diseases of the Joints.* By B. C. Brodie, v.p.r.s., &c. Third Edition.—*London*, 1834.

2. *The Cyclopædia of Anatomy and Physiology*, July.—Articles: *Elbow, Joint of the; Abnormal Anatomy.—Hip-joint, Abnormal Conditions of.—Knee-joint, Abnormal Conditions of.* By R. Adams, Esq. —*London*, 1837-8-40.

3. *Cyclopædia of Practical Surgery.* Part IV. January.—Art. *Articulations.* By J. Blackburn, Esq.—*London*, 1840.

4. *Dissertatio Medica Inauguralis de Cartilaginum Articularium ex Morbis Mutatione.* Auctore L. H. Schumer, Jun.—*Groningæ*, 1836. 8vo, pp. 64.

*Inaugural Medical Dissertation on the Alteration of Articular Cartilages by Disease.* By L. H. Schumer.

4. *De Fungo Genu necnon de Tuberculis in hoc Morbo inventis.* Dissertatio quam deft. F. J. Lederle.—*Petropoli*, 1838. 8vo, pp. 81.

*On Fungus of the Knee, and on the Tubercles connected with this Disease.* By F. J. Lederle.—*Petersburg*, 1838.

The works whose titles we have prefixed to this article will rather indicate the object than form the subject of our remarks. The first, that of Sir Benjamin Brodie, is above the need of a reviewer's praise, having already taken its just place in medical literature among the very best works of the last century. We have already (Vol. VII. p. 535,) given some account of Dr. Schumer's Essay. The Article by Mr. Blackburn evinces an extensive acquaintance with the writings of the best authors on the subject, and a judicious appreciation of their opinions; and those of Mr. Adams have much of the same practical excellence which has distinguished all his contributions to the Cyclopædia of Anatomy. In this respect his writings stand in strong contrast to several of the German dissertations with which we have compared them, and which, though possessing some pathological interest, as representatives of the views entertained in Germany on these subjects, might have been written by one who never inspected a human joint in a state of disease.

Our object will be to afford a succinct view of a part of the present condition of knowledge in the pathology of joints; to point out some of the general principles of disease which their morbid changes illustrate; and by the application of other general principles of pathology deduced from the examination of other diseased organs, to endeavour to remove some of the difficulties which at present obscure their explanation. Our task will be at once more satisfactory and more easy, because the diseases of joints have now, both from their importance and their pathological interest, received an unusual share of attention from some of the best surgical writers; and there is perhaps no system of organs whose diseased changes admit of more complete illustration. There is certainly no part of the body by which more important evidence is afforded of the varied progress of disease. Composed as they are of a great variety of tissues, —each of which, although it may sometimes be separately affected, is in some degree dependent on the rest—and peculiar in their form and arrangement, and functions, the joints in their diseases present a pathological microcosm, and afford a singular opportunity of studying the effects of the same morbid processes as they are modified by peculiarities of tissue, of construction, and of vital properties.

1. *Diseases of the Synovial Membranes.* The synovial membranes are arranged on the same general plan as the serous, forming like them closed sacs. That they pass over the surfaces of the cartilages can no longer be a subject of doubt, since, in addition to the previous strong and almost sufficient evidence in favour of that view, Henle has found, with the microscope, that a fine cellular corium covered by a delicate layer of epithelium passes over the exposed surface of all the articular cartilages.* There is undoubtedly also "some analogy in the diseases" of the synovial and of the serous sacs; but it is far from perfect; and on the very first point which Sir B. Brodie mentions (p. 7), that of "swelling of a joint from a preternatural quantity of fluid collected in its cavity, without pain or inflammation," we find an important difference between the two. The dropsies of serous sacs are of two distinct kinds: in one the fluid passes out from the vessels by a power which is entirely local, by an act of secretion; in the other it transudes from them under the influence of simple mechanical pressure, which may be imitated at will by artificial injections, or its excessive effusion is the consequence of some physical change in the condition of the blood. In the first class of effusions we find the effects of pleuritis, peritonitis, and more definitely, hydrocele; in the second, the ascites of obstructed partial circulation, and the more general dropsy of obstructed circulation from disease of the heart, in which anasarca of the cellular and other soft tissues coincides with effusion into the serous sacs.

Now the synovial sacs are subject only to the first kind of effusions; to those which arise from a local secretory power. It has often been observed as a distinction between serous and synovial membranes, that the one are commonly, the other never, the seat of simple dropsical effusions, and the difference has usually been ascribed to some yet unseen diversity of tissue, or to a yet more vague peculiarity of vital action. We believe that the cause of the distinction lies in neither of these circumstances; but in the peculiar construction of the cavities which the synovial membranes line, and in the difference between them and most of those that are lined by serous membranes. As the blood passes along the vessels in healthy circulation, just sufficient fluid transudes through their walls to keep the adjacent tissues and surfaces slightly moist; but if the pressure of the blood against the walls of the vessels be increased, by any obstacle to the passage of venous blood, or (which comes to the same thing) by an increased force of the arterial blood without any increased means for its passage through the veins, more fluid must of necessity pass through the walls of every vessel that is not supported by the unyielding pressure of some firm substance around it. In all the soft and easily extending tissues therefore, and in all the cavities with yielding walls, dropsy is thus at once produced, the force of the obstructed blood being amply sufficient to distend those parts. But it is different

* Ueber die Ausbreitung des Epithelium. Müller's Archiv, 1838, Heft 1.

with the hard tissues, and the unyielding cavities, such as the cartilages, the tendons, the cerebro-spinal cavity, and the joints; in these no mechanical dropsy (such as arises from disease of the heart) can take place, for the same force by which the fluid is effused in increased quantity in the parts already mentioned, is totally unable to distend them. Hence, though all the tissues around them are soaked in fluid, no tendon or cartilage is ever anasarcous in a general dropsy; and this not for want of vessels, but because the walls of those vessels are bounded by unyielding tissues into which fluid could transude only under a far greater force of pressure. For the same reason, while in similar cases the abdomen and the pericardium are distended, and the lungs are compressed by the fluid effused from the obstructed vessels within and around them, the nervous centres in their cavity of fixed and unalterable size, and the joints with their tough unyielding walls, are unaffected.

The dropsies of synovial sacs, then, are entirely the result of a local act of secretion; they correspond to hydrocele, and to the effects of pleuritis and peritonitis, &c.; but there are no affections of the joints analogous to ascites or the coincident effusion from obstruction into other serous sacs. In passing, we would point out these facts as illustrative of the difference between secretion and mere mechanical effusion; and of the varieties of the forces by which, under different circumstances, fluids are made to pass through the walls of vessels. The force of secretion is in these cases shown to be sufficient to produce, in a few hours, such a distension of a joint as could not have been effected in as many years by the force of exudation exercised by an obstructed column of blood driven on even by an hypertrophied heart.

But this affection, this hydrops articuli, in which an increased quantity of fluid is poured into a joint without pain or inflammation, and in which the fluid differs but slightly from common synovia, is of rare occurrence. The more frequent cause of effusion of an increased quantity of fluid into a joint is a distinct inflammation of the synovial membrane.

On reviewing the cases of this class which Sir B. Brodie relates, as well as those that have fallen under our own observation, it is evident that as subjects of morbid anatomy the changes produced by this inflammation present peculiarities of two kinds—one depending on the peculiarity of the tissue itself, the others on that of its construction and arrangement. The latter have been by no means sufficiently considered, though they are of great importance in explaining some facts which, at first sight, appear anomalous.

As the subject of disease the synovial membrane cannot be abstractedly considered. Regarded as a homogeneous thin layer of tissue lining the whole joint, it is yet evident that both in health and disease the performance of its functions must be materially influenced by the character of the tissue subjacent to it, and from or through which it receives its vessels. Sir B. Brodie (page 6) states it as a principle to which all will agree, that "living organs are more subject to have their natural functions deranged in proportion as they are more vascular, and as they are employed in a greater degree in the process of secretion." But the degree of vascularity in the synovial membranes of different joints, and even in that of the same joint, is by no means fixed; the same membrane where it lines the articular cartilage is far less vascular than where it lines cellular tissue or fat; less vascular, again, where it is placed on ligament than where it lies on bone or periosteum. The liability to inflammation, and the severity of the results of the same inflammation of a synovial membrane, will therefore be determined not by a certain vascularity peculiar to itself (as they are in muscle or in bone), but by the vascularity of the tissues beneath it; for the vascularity of the various portions of the synovial membrane is determined by that of the tissue through which each receives its vessels.

We might illustrate this principle of the influence of subjacent tissues upon the diseases of lining membranes by several examples; but we will take only one, that of the inflammation of arteries and veins. The lining membrane of the whole system of blood-vessels is continuous and in all its parts similar, whether we receive the older view of an internal thin layer of cellular tissue or the correct demonstration of Schwann, that it is chiefly composed of fibres of elastic tissue, in which, if any, a longitudinal direction is discernible, and whose surface is lined by a fine epithelium.* In either case the linings of the arteries and veins may be considered identical. Yet how different are their diseases. Acute arteritis is one of the rarest affections, but acute phlebitis is of comparatively common occurrence. And in the effects of each there is no less marked a difference; in arteritis the highest degree of inflammation produces only a slight effusion of lymph or adhesion; while in phlebitis, suppuration, and the abundant formation of lymph are common and almost constant effects. There seems no other sufficient reason for such a difference in the results of the same disease in two tissues of the same kind than this—that in the one the tissue receives its vessels from a surrounding very vascular layer of cellular membrane, in the other from a layer of elastic tissue, in which vessels are but just demonstrable; and that in each part its own vascularity is directly proportioned to that of its subjacent tissue.

Other examples of a similar kind will at once present themselves to the reader's mind. To apply the principle which they establish to the affections of the synovial membrane: The tissues on which it is placed might be arranged according to their vascularity in the following order: adipose and cellular tissue; bone and periosteum; tendon and ligament; fibro-cartilage; articular cartilage; and as a general rule (to which, however, certain local circumstances will cause individual exceptions,) we think it will be found that the morbid changes of the several parts of the membrane lining these tissues, both in severity and frequency, follow the same series.

When, for example, we examine an old diseased knee-joint, in which the history shows that inflammation commenced in the synovial membrane, we do not find its whole lining equally affected; but by far the most severe results of the disease are seen on the portions beneath which adipose tissue is placed, as above and by the sides of the patella; here it is most thickened, most spongy, most vascular, and covered by the greatest quantity of lymph; on the periosteum around the edge of the cartilage and on the front of the crucial ligaments, similar effects are much less in

* Encyclop. Worterb. des Medicin. Wissenschaften. Art. Gefässe. His view is confirmed by Gutenberg, Raeuschel (De Arter. et venar. structura), and Henle, (Ueber die Ausbreitung des Epithel. Müll. Arch. 1838,) though in some measure contradicted by Valentin, (Repertorium, &c.)

degree; and on the surface of the cartilages they are altogether absent. But the synovial membrane is over all these parts, *per se* the same; and there is every reason to believe that all of it is simultaneously and equally attacked by the disease; so that there can scarcely be any other cause for the differences than the different natures of the subjacent tissues.*

In like manner (though in the determination of this result there must be many more modifying circumstances), we believe that the comparative frequency of disease of the synovial membrane in different joints, depends in great measure on the degree of vascularity of the tissues by which the walls of each joint are formed. In none is the disease so common as in the knee-joint, and no joint has more of its synovial membrane in contact with adipose tissue; next to the knee in this respect may probably be placed the elbow, and here again a considerable quantity of loose and vascular tissue surrounds the joint; while in the hip, where there is a complete and dense fibrous capsular ligament, with the low degree of vascularity peculiar to that class of tissues, primary disease of the synovial membrane is by comparison of very rare occurrence. Sir B. Brodie regards the exposure to cold as the chief cause of the frequency of inflammation of the knee-joint, and Mr. Adams partially coincides in that opinion; the influence of cold cannot be denied, but its sufficiency may be doubted when the disease is far more rare in the wrist and ancle, which are more exposed to cold than the knee, but a greater portion of whose contour is occupied by fibrous tissues.

These circumstances, then, which are often regarded as anomalies, namely, the varied frequency of common inflammation of the synovial membrane in different joints, and the varied degrees of its severity, as exhibited in its products, in different parts of the same joint, are, we believe, fairly explained by the different degrees of vascularity possessed by the surrounding subjacent tissues, from which the synovial membrane receives its vessels. We may now proceed to the diseased appearances whose peculiarities are more probably referrible to the nature of the synovial tissue itself.

The effect most commonly and often permanently left by inflammation of the synovial membrane is thickening. This differs from the analogous change produced in a serous or mucous membrane, by the parts being less dense and firm; the thickened part is soft and easily tears; it has a spongy œdematous character; its free surface, instead of being smooth and polished, acquires a delicately granulated or almost villous aspect, not unlike that of a granular conjunctiva; and at the edges of the cartilages it often overlaps them, just as a chemotic conjunctiva overhangs the margin of a cornea.†

In this condition the tissue of the membrane undergoes no material change; it is only swollen by the effusion of serous fluid and usually of

* By this explanation, too, the chief objection urged against the presumed passage of the synovial membrane over the cartilage is destroyed; if the cartilages themselves are obscure in their manifestations of disease, so must be the membranes which are so intimately connected with them, and which receive from them a great part of their vascular supply.

† In this as well as in many other circumstances, in both their natural and morbid structure, one cannot but be struck with the closeness of the analogy between synovial membrane and the conjunctiva. The comparison was first made by W. Hunter, who was, indeed, in this part of his descriptions, singularly felicitous; witness the famous comparison of the serous sacs to double nightcaps.

lymph into its areolæ, which, if the affection be not complicated with other articular diseases, are often rapidly removed after counter-irritation or other means. There is another state which possesses some, though an obscure, relation to this simple thickening, but which is much more serious in its nature. Sir B. Brodie regards it as a change peculiar to the synovial membranes of joints, and belonging to the same class with tubercle, carcinoma, &c.

"The morbid action evidently originates in the synovial membrane, which loses its natural organization and becomes converted into a thick pulpy substance of a light brown and sometimes of a reddish-brown colour, intersected by white membranous lines. As the disease advances, it involves all the parts of which the joint is composed, producing ulceration of the cartilages, caries of the bones, wasting of the ligaments, and abscesses in different places." (p. 79.)

This form of disease is that which Dr. Lederle, with most of the German writers, describes as fungus genu, and to which he applies the extraordinary appellation of "dynamico-vegetative pseudoplasm." It is also one (and if they understood anything definite by the name, the especial) form of disease described by the old writers as white swelling. We are inclined to think that it is more commonly an effect of simple chronic inflammation of the membrane than Sir B. Brodie regards it; its most common situation, the knee, is that in which the synovial membrane is far most frequently inflamed; its history is often that of protracted inflammation; the shades between it and common thickening are so numerous that they are scarcely separable; and it does not, like other specific or malignant diseases, affect either the same tissue in different parts or different tissues coincidently. Further pathological investigation is still necessary on this subject; the interest of which is the greater because the disease in its advanced stages admits neither of cure nor alleviation; and if it be in any case the result of the neglect of a more simple disease, its prevention should of course be the object of much greater care than is usually bestowed on common stiff joints.

In thickening of synovial membranes, serum and lymph are effused in the areolæ of its tissue, and very generally in the adjacent parts, which become hard, tough, and unyielding, lose all their power of sliding upon one another, and in some cases form an almost homogeneous, brawny, infiltrated mass, in which the dissector has difficulty in discovering even those things that in health are among the most prominent. At the same time lymph and serum may be secreted on the free surface of the synovial membrane and into the cavity of the joint. The effusion of lymph, however, is on the whole less frequent in the synovial than in the serous sacs; a circumstance in which again they appear in some measure intermediate between serous and mucous membranes. The lymph may form adhesions between the opposite surfaces of the membrane; for adhesions are the result of inflammation, not of any particular tissue, but of all tissues which are so arranged as to present two surfaces sufficiently near each other to permit the lymph effused upon one to adhere to that upon the other, and thus in one mass with it to be organized. Where this arrangement is wanting, whatever be the tissues inflamed, it is evident no adhesion between them can take place. In the synovial sacs adhesions are on the whole less frequent than in the serous; probably in consequence of the little power which some of the tissues composing the former possess of producing or receiving new vessels. Thus, suppose lymph effused on the surface of a synovial membrane opposite a cartilage; the chance that the cartilage will either produce or receive new vessels to communicate with those that form in the lymph is but slight. But there are only a few parts of the joints in which the synovial membrane covering one tissue is not opposed to that covering cartilage, and the adhesions that do form are generally found in these parts, as, for example, where the synovial membrane of the wall of the joint is opposed to its own reflexion over the bone. Hence in the knee, the frequency of the cases in which the cavity of the articulation is obliterated above the patella, and at the sides of the condyles of the femur, and round the head of the tibia. The cases are rare in which adhesions to pass the surface of the synovial membrane covering the cartilages: but they occasionally happen and are, we think, more common in the ancle than in any other joint. There are several specimens of the kind in the museum of the College of Surgeons; and we have seen more than one in which a distinctly vascular adhesion passed from one cartilage to the other without having any connexion with the more vascular parts of the synovial membrane lining the walls of the joint; evidence which alone affords as good proof of the vascularity of cartilage as most *facts* in medicine are based upon.

The next degree of the effects of inflammation of the synovial membrane is suppuration. It affords a good example of the production of pus without ulceration; though the doctrine of suppuration from a sound tissue has lost its verbal truth since Henle proved the existence of a synovial and a serous epithelium, and rendered it probable that before pus-globules can be produced from a membrane's surface, the epithelium cells must be removed. The pus is therefore produced from the *raw* surface of the synovial membrane, just as it is from the cutis when its epidermis is removed. The production of pus, like that of dropsies in synovial sacs, is by a process of secretion, which overcomes all the resistance of the walls of the joint, and often distends them to an enormous size. In this case there can be no doubt of the propriety of Sir B. Brodie's advice that the fluid should be let out by an opening made into the joint; the sufferings which the patient endures from an otherwise irremovable and still increasing collection of fluid distending all the tissues around it, at once declare the necessity of the measure. He limits the practice to these cases; and except in considerable collections of pus we cannot but doubt the propriety of cutting into a joint. Yet there are many good practitioners who do not hesitate to adopt the same means in many other cases, under the idea that the inevitable anchylosis will be accelerated. Their arguments for this practice, however, seem to furnish their own contradiction: it is true that anchylosis often follows the wound of a healthy joint, and so does death sometimes; but, say they, the opening of a diseased joint is very different from opening a healthy one, it produces no ill effect, and may be done with perfect safety. But if it produces no ill effect, will it produce anchylosis, which is the most common ill effect that follows it when performed on a sound joint? For what are the obstacles to the occurrence of anchylosis in the common course of the disease which the operation removes? We believe none—unless the opening lets out such a quantity of fluid as was sufficient to separate the surfaces that should adhere. For osseous anchylosis to take place, the cartilages must be removed and the bones exposed; granulations must arise from each of them, and uniting become osseous; if pus or any other fluid keep the granulations apart and prevent their union, it should undoubtedly be removed; but if not, the process will go on as plainly as the union of a simple fracture, and, for aught we can see, it would be as wise to pass a lancet between the ends of a broken bone, under the idea of accelerating their union, as to open a joint not full of pus in the hope of accelerating its anchylosis.

If the pus be not artificially removed from a joint, it will slowly make its way out by ulceration through one of the spaces between the dense tissues of the walls, or if the joint have a capsular ligament, through the meshes of its fibrous fasciculi. The aperture first forms where the resistance is least, in accordance with common physical laws; the fluid pressing equally in all directions distends and produces its injurious effects most rapidly where the parts are weakest. The artificial opening of the distended joint is thus the more advantageous because it is a rare coincidence in which the weakest is at the same time the most dependent part; and a naturally formed opening will seldom be placed so as to permit the fluid to pass off by it rapidly enough to keep the joint empty. Thus in the knee, openings most frequently form at the sides by the lateral ligaments; or in front just by the sides of the ligamentum patellæ; or if they form behind, it is too often by the pus creeping through a long and tortuous course, in the ham and down by the heads of the gastrocnemii. An artificial opening may of course be made wherever the patient's position and other circumstances render it most desirable; cæteris paribus, that which Sir B. Brodie recommends, in the most depending part, will be the best.

That important morbid condition in which pus is simultaneously deposited in a number of joints is still, as to its pathology, most obscure. Scarcely any advance has been made in our knowledge of it since Mr. Arnott's admirable paper on Phlebitis was published in the Medico-Chirurgical Transactions. Of its frequent coincidence with phlebitis there can be no doubt; but it is far from proved to be a consequence of it: on the contrary, cases are not wanting in which the venous system, though carefully examined, has been found quite sound. On all connected with these cases much more evidence is yet required; and it is to be hoped that their explanation will be one of the first practical results of the microscopic observations on pus and suppuration, which are now occupying so many active minds, but have as yet been lamentably barren of profitable results.

In the natural progress of disease the pus formed in a joint will pass out of it by ulceration; it is much more rare for it to pass from without inwards, and by ulceration to produce perforation of a joint. But such cases (though as yet, we believe, almost undescribed,) do occur, and they are perhaps the most terrible in their symptoms and progress of all the diseases that affect the joints; for they are equal to the ulcerative perforations of the serous sacs in their rapidity of local progress, and though less quickly fatal, are even far more painful than they are. Happily the conditions in which they can occur are but few; they are only those in which a collection of pus is formed in some tissue adjacent to a joint, and is prevented from making its way externally by some dense unyielding substance between it and the surface of the limb. This is sometimes the case when the head of a bone is necrosed, or has a suppurating cavity formed within it; as the fluid increases, and the bone around it is removed, it may at last perforate the joint through the ulcerated cartilage, and the destruction that follows it is alike rapid and painful. More rarely, an abscess formed externally to the joint opens into it at some part where the synovial membrane is guarded by weaker tissues than those which lie superficially to the pus, and in these cases also, a rapid destruction of cartilage, with intense inflammation of the synovial membrane and the most acute suffering are sure consequences. Amputation, which the severity of the symptoms at once demands, probably affords the only prospect of recovery.

We have considered the several degrees of the affection of the synovial membrane separately, because it is of the utmost importance in their treatment to know that in the early stages of disease of the joints only this part may be affected, and that, therefore, the limb may by timely and proper care be restored to complete health. Sir B. Brodie particularly insists on the necessity of knowing what the early conditions of these diseases are, before they have proved fatal to the limb or life by involving all the tissues adjacent to that primarily affected. It is probably from lack of opportunity of observing these early changes, that nearly all writers but himself describe the affections of joints as so complex and so rarely isolated in any one of the tissues composing them. This is in some measure the case with Mr. Adams, who, though not denying the probability that the diseases of a joint may commence in any one of its tissues separately, yet refuses to take advantage of the evident distinctions that might be founded on that fact, and writes of inflammation of the hip-joint (*arthritis coxæ*) in general, distinguishing only the three forms of acute, strumous, and chronic rheumatic inflammation; and of the diseases of the knee-joint, under the general name of arthritis genu, distinguished only into acute, simple chronic, chronic rheumatic, and chronic strumous arthritis. This, however, is depriving morbid anatomy of much of its practical benefit. To be sure, when we get a diseased joint to look at, it is in general only when it had ceased to afford any hope of ever being useful to the patient, and when he is either dead or maimed; and then no doubt we may find the ravages of synovitis, chondritis, osteitis, and many more such enemies as formidable in nature as in name; but we should remember that there probably was a time when there were fewer to contend with; that all these crept in one by one, each establishing himself on the ground near that which was already occupied by one of his allies. If anything is to be done for the cure of this class of diseases it must be attempted before many of the tissues are affected; the chances of benefit are always inversely to the number of those terrible *ites*, and the more separately we consider them all the better shall we be prepared to combat with each.

II. *Diseases of the Articular Cartilages.* We pass now to the consideration of the morbid alterations of the articular cartilages, and we come at once upon a fundamental question: Are there any proper diseases of articular cartilage? Are not all those that are so called the results of disease of the adjacent tissues and of the action of their products?

The doubtful circumstance on which the answers to these questions must ultimately depend is the organization, or, to speak more definitely, the vascularity of the cartilages. We will first consider the statements and opinions of those who oppose the vascularity and independent morbid alteration of these tissues, and then express our own opinions and those to which we assent.

The work of Dr. Schumer affords a good summary of the arguments generally advanced by the opponents of the independence of cartilages. The vascularity of articular cartilage might, as he rightly says (p. 11), be determined, 1st, by the flow of blood from a wound of its substance; 2d, by injection; 3d, by microscopic examination; 4th, by the morbid changes, if they are of the kind which cannot occur without vessels. But by none of these means, he and many others affirm, can their vascularity be demonstrated.

That no blood flows when an articular cartilage is wounded we readily admit; but no blood flows when the iris is cut, as in making an artificial pupil, or in any other operation, unless it is torn away from the ciliary ligament. Yet who doubts the vascularity of the iris, or that it is subject to peculiar diseases? And the tissue of articular cartilage is even more adapted for the prevention of hemorrhage from vessels divided in it than that of the iris is; for it is compressed within the limits of the space which by its unrestrained elasticity it would occupy, and, therefore, when it is cut its particles instantly spring up against each other, and at once close any vessel that may be divided. The evidence of the absence of bleeding from its wounds is therefore valueless; the hemorrhage from a tissue is not directly proportioned to the size of its vessels, but to the circumstances in which they are placed, whether the tissue around them is unyielding or loose, whether and how soon they can contract, &c.

The evidence of injection is equally unsatisfactory. It is, we believe, true that no one has yet succeeded in forcing a coloured fluid into the very substance of the healthy articular cartilage; but it may be driven into vessels running over its free surface. We have done this in a perfectly healthy joint; but it is much less difficult when, by an inflammation, the synovial membrane covering the cartilage is slightly swollen; injection will then pass in distinct branching vessels derived from the circulus articuli vasculosus, for more than half an inch over the edge of the cartilage, in exactly the same manner as the vessels pass from the conjunctiva over the front of the cornea. Now it is not in the smallest degree probable that these vessels stop suddenly at this distance upon the surface of the cartilage, without passing like those of the cornea (to which they are in other respects so similar) over the whole surface, and dipping into the substance of the cartilage itself. Nor is it more probable that a dense non-vascular tissue should thus be placed between two vascular surfaces; there is but one instance in the body of a non-vascular organ so situated—the crystalline lens—and we need not say how distant is the analogy drawn between it and an articular cartilage.

In addition to these vessels of the synovial membrane covering the cartilage, we believe that another set exists which pass vertically from the bone through the little canals, which are so distinct by the pink points that are seen when a slice is cut from the surface of the cartilage of a young animal. These have not indeed been yet injected; but the art of injecting is not yet so perfect that from its negative evidence we can have any right to draw a positive conclusion. The vessels of the healthy cornea have been demonstrated by injection only within the last four years; those of the cartilages of the ribs and larynx within the last ten years; and those of the tendons have only been injected within the last twelve months. The articular cartilages evidently present many more difficulties to injection than these; but there is no reason to believe that they may not yet be surmounted. Till the tissues just mentioned had been injected, their vascularity was denied or doubted by those who are not content with any but visible proofs; but long previously, their morbid changes were sufficient to establish their vascularity to every impartial mind.*

That vessels should be invisible when the articular cartilages are examined with the microscope is no wonder; they cannot when empty be discerned by it in many even of the most vascular tissues.

We come, then, lastly, to that which is by far the most interesting and important argument for the existence of an independent morbid action in the cartilages, and that such an action as we cannot conceive to be carried on without blood and blood-vessels—the changes which they undergo in disease. This subject has been examined experimentally, and especially by Dörner,† Gendrin,‡ Cruveilhier,|| and Schumer, and the general result of their investigations is the impossibility of exciting any evident inflammation in an articular cartilage by any mechanical injury. But it is clear that these cases furnish of themselves the contradiction to the conclusions drawn from them; for no effect was produced by the injury upon the cartilage, although all the other tissues of the joints were vehemently inflamed. Now, if the cartilage were not destroyed by the action of its own vessels, why was it not destroyed by those means to which they ascribe its destruction in common inflammations of joints? The experiments show that in the midst of a most acute inflammation of the synovial membrane, such as is produced by cutting a joint wide open, the cartilages remained for some days unaltered. They, therefore, prove nothing; for they are as strong evidence against the power of any external means produced by synovial inflammation to destroy the cartilages as they are against the cartilage being able to destroy itself. They only prove that in an acute inflammation of a joint the cartilages are not always ulcerated.

But suppose that by disease or after an experiment the cartilage is removed from the head of a bone. What are the means by which that removal has been effected? Softening and solution in the pus or other fluid effused into the joint? Absorption by the surrounding synovial membrane, or a tissue produced from it on purpose? Or independent ulce-

* Since this was written our belief has been most happily confirmed by Mr. Liston's account of his injections of the articular cartilages of diseased joints, in a paper read at the Medico-Chirurgical Society. Of course we can at present only announce the fact; but we may add that an inspection of the specimens that were exhibited in the Society's rooms unequivocally established the author's description of vessels in the very substance of the cartilage. The injections were among the most beautiful we have ever seen; and we cordially congratulate their possessor on having surmounted the greatest difficulty of this art.

† De gravioribus quibusdam cartilaginum mutationibus. Tuburg. 1798.

‡ Histoire Anatomique des Inflammations.

|| Archives Générales de Médecine. Fevr. 1824.

rative action in the cartilage itself? The first is the common opinion of those who do not receive the third—the second is that propounded by Mr. Key*—the third is that of Sir B. Brodie and many others, and that which we have now little hesitation in adopting. The first two explanations can be applied only to those cases in which the cartilage is progressively removed from its synovial surface towards the bone; they can have no application in the cases in which its removal is effected in the opposite direction, in which it is, as it were, undermined and its connexion with the bone destroyed. In these latter cases it is by all supposed to be absorbed by granulations produced from the bones; but we shall offer some reasons for doubting the correctness of this view.

First, for the theory of solution in pus. Schumer relates an experiment in which a portion of cartilage, placed in a suppurating fistulous passage, was found after seven days opaque and yellowish, and half dissolved. But ten such experiments could have no weight against the fact which every one must have had the opportunity of observing, viz., that after pus has remained even for many days in a joint, the cartilages may still be neither softened nor in any other way altered; and that if they are irregular in certain isolated parts of their surface, the parts which still remain attached to the bone are quite firm and polished. This is especially the case in that class of suppurations which occur coincidently in several joints, and which we have already alluded to as sometimes accompanying phlebitis. In these cases, after a joint has been distended for many days with pus, all the evident change in the cartilage is that a portion seems as if it had been chipped out of it with some blunt instrument. The surface that is left, and all the rest of the cartilage is still firm, of its natural bluish-white colour, and intimately attached to the bone. Now, if pus had the least power of dissolving cartilage in the living body such facts as these could never be observed; the cartilage in such a suppuration of a joint would be evenly thinned over its whole extent, and that which remained would be soft and loose and half dissolved, or flocculent and unable to bear the pressure of the finger; but such an appearance is in common inflammation of a joint never seen. Portions of cartilage are always entirely removed; and that which remains possesses a healthy aspect if not its normal connexions.

The theory of Mr. Key, which is in part adopted also by Mr. Adams, is far more ingenious and is founded on facts of more seeming authority, than this of solution by pus. An admirable criticism of it forms the greater part of the important appendix to the present edition of Sir B. Brodie's treatise. The theory is briefly this: When ulceration of the cartilage ensues as a result of synovial inflammation, the removal of its tissue is effected not by its own vessels but by those of a very vascular process of synovial membrane which grows over the border of the cartilage, or by those of a similarly vascular membrane produced from the synovial membrane and receiving its vessels from it, which spreads over the free surfaces of the cartilages and absorbs them.†

* On the Ulcerative Process in Joints, Medico-Chirurgical Transactions. Vol. xviii.

† We cannot but remark the singular mode of expression which Mr. Key, reasoning straightway from facts to final causes, adopts in reference to this process; speaking of it as a curative effort of nature, whose object is in these diseases of the joints to produce, as a natural *cure*, anchylosis. This may be surgically speaking a cure, because

The fact upon which this theory is founded is that in many cases where the synovial surface of a cartilage is grooved, or as Sir B. Brodie happily expresses it, *chiselled* out by ulceration, the depression is found occupied by a process of membrane from its synovial lining, which has a very vascular and fringed border, like one of the "glands of Havers." This Mr. Key believes has absorbed the cartilage by a process analogous to that by which the removal of the sequestrum of cylindrical bones under necrosis takes place. Now the question of the absorption of dead bone is too long to be entered upon at length here; but we may observe that the experiments of Mr. Gulliver, supported as they are by many facts of constant occurrence, render it certain that a sequestrum separated from its vascular connexion is never in any degree absorbed by the surrounding vascular parts, but remains, as the loose fragments of fractures often do, unaltered for months and years. The same experiments render it very doubtful whether a dead portion of bone even still adhering to living bone, is ever lessened by the absorption of the vessels of another part; much less then is it probable that a living tissue, however slightly vascular, should be absorbed by the vessels of another adjacent tissue; nor do we know a single example in which it can be proved that the vessels of one living part or tissue have the smallest share in effecting the ulcerative removal of another. It is evident, therefore, that no imagined analogy to any of these other processes is deducible in favour of Mr. Key's view, for they are themselves, if not opposed to his idea, at least most ambiguous.

Again, it is clear that this assumed mode of ulceration by the action of a destroying growth from the synovial membrane cannot explain all cases; for in many there is no such growth; in many the cartilage is removed when it was opposed to another cartilaginous surface, and in these and in many more the ulceration occurs in a part of the articular surface to which no synovial growth could have access, as on the very centre of the patella, on the middle of the condyles, and on the head of the femur not near the ligamentum teres, on the middle of the head of the humerus, &c. From all these parts portions of cartilage are often found removed while that around them is healthy; so that it is evident that even in some of the cases in which the membranous process does exist, it could only have arrived at the ulcerated part by passing over the healthy surface. But in many of these cases of isolated ulceration, and indeed in the large majority, the synovial membrane has no processes growing from it at all. And it is not in the least probable but that in all these cases the process employed is essentially the same; a single case, therefore, in which a growth from the synovial membrane is not found, though the cartilage is absorbed on its synovial surface, would throw great doubt upon the theory; and when such cases are far from rare they must be fatal to it. They are so far from rare that we believe that many surgeons to whom the ulceration of the cartilages is a familiar appearance have never yet seen such processes from the synovial membrane as Mr. Key describes.

But still this vascular growth is sometimes seen exactly fitting into the patient does not die—yet we scarcely call it a cure, if from inflammation of his eyes a patient loses his sight; and vision is not more the function of the eye than motion is of a joint.

the cavity in that portion of the articular surface from which the cartilage is removed, and certainly to an imaginative mind it looks just as if it had been eating and growing on the food it derived from the edges of the hollow it has made; it looks like a worm in its hole. How then did it get there? To explain it the structure of the joints must be considered; they are cavities with firm and scarcely yielding walls, and they admit of no change of volume except with an inversely proportioned alteration in the quantity of their contents. If the cavity of a joint increase in size, more substance must pass into it to fill it; if it decrease in size something must pass out of it. They are in this respect exactly analogous to the cranium, into whose cavity, if the mass of the brain become smaller by atrophy or other loss of substance, more fluid must be and always is effused; if the brain become larger, fluid must pass out of the cranium (unless in children in whom the head can expand) and the brain is then found dry and without fluid either in its ventricles or membranes. There are cases of cerebral disease in which effects are observed exactly analogous to this growth of the synovial membrane into the joints. When, in consequence of an apoplectic effusion, an abscess or any other disorder in which a portion of the brain is destroyed, the volume of the contents of the cranium is lessened, inasmuch as the size of its cavity remains for the time unaltered, it is certain that either something must be added to the volume of the contents or that the volume of the cavity must be reduced. The contents of the cavity must exactly fill it or a vacuum would exist, which we need scarcely say is impossible. The subject is admirably illustrated by some of the cases of atrophy of the brain detailed by the late Dr. Sims, in his paper on that subject, in the 19th volume of the Medico-Chirurgical Transactions: "The chasm occasioned by the atrophied state of the brain," he says, "we observe to be sometimes filled up by serous fluid and by *deposits of bone on the skull*"—"the hypertrophy which takes place in the bones of the cranium is very frequently confined to the inner surface of the os frontis, in other cases it is more general." (p. 367.)

There can be no doubt that in the living body as in inorganic matter, if the size of an air-tight cavity be increased its contents must expand, or if not expansible must have something added to them to fill the cavity; or if the size of the contents of a cavity be diminished its walls must fall in. These are the simple principles on which when in the adult the brain grows larger the skull grows thinner; and when the brain becomes smaller the skull becomes thicker, or more fluid than natural is effused within it. To apply the same principles to the disease of the joints now under consideration; a portion of the synovial surface of an articular cartilage is removed by the ulcerative process of its own vessels; what is to fill the space thus produced in the cavity of the joint? In most cases, as in most of atrophy of the brain, fluid (either pus or synovia) is effused in increased quantity; but in some, as in some of atrophy of the brain, the surrounding tissues grow in and fill up the chasm. The process, or as it might be called the hypertrophy of the synovial membrane, is the exact analogue of the hypertrophy of the bones of the skull.

In this explanation nothing improbable is involved. It is assumed that the first change is the absorption of the cartilage, and that it may be the first is certain because in some early examined cases it is the *only* change; by such an absorption a vacuum *must* be formed in the joint; and something *must* fill it up; that *something* is either fluid or an increased growth of synovial membrane or a newly-formed false membrane. We can indeed determine, if the part of the cartilage absorbed be known, what will be produced to fill its place; if the middle of the surface be ulcerated, fluid will be effused and no membrane produced; if the borders of the cartilage, then membrane grows into the cavity as fast as it is formed.

Thus much of the presumed explanations that have been offered of superficial ulceration of articular cartilages, to remove the apparent difficulty of their possessing too small a degree of vascularity to effect their own ulceration. Neither of them is free from far greater objections than those which they are intended to surmount.

The opinion is more commonly received and certainly more plausible that in the deep ulceration in which the cartilage is undermined from its connexion with the bone, the vascular granulations that grow into the hollow between them are the agents by which the absorption of the under surface of the cartilage is effected. But even against this view there are many objections; as the improbability of one tissue being absorbed by the vessels of another, and that healthy bone (for in some though not in most of these cases the bone is found unaltered) should produce granulations to do such irreparable mischief. It is certain that if the absorption of the under surface of the cartilage were the first step in the process granulations or something *must* grow from the head of the bone to fill up the cavity that would else be formed; and that the cartilage is first absorbed is rendered probable by the frequent coincidence of similar absorption on its opposite (synovial) surface, which we have already shown is often a primary affection.

The growth of granulations into such a cavity as the independent ulceration of the under surface of the cartilage would form is easily producible at will. If a hole be bored in a bone and the integuments replaced over it for a few days and then again raised, the hole will be found exactly filled by a growth of vascular granulations from the surface of the tissues that lay over it, just as in this case each little cavity on the under side of the cartilage is occupied by a growth of granulations from the subjacent bone.* The same action no doubt produces the granulations in both cases, as well as in all those in which granulations appear to be eating away the surface of an ulcerating tissue. In all the growth and increase of the granulations is secondary; a necessary consequence of the removal of adjacent tissues; they do not form the holes, but they grow into the holes that are formed by the tissue's own vessels.

The explanations, then, which the opponents of the independent morbid action of the cartilages have offered are at present insufficient to account for the phenomena. The removal of cartilage is fairly referrible only to a process of ulceration which is effected by its own power; and even in the absence of any other evidence for their vascularity this alone might be sufficient proof of it. When to the evidence which it affords we add the proof of the vascularity of their synovial surface,—the occa-

* This experiment was often performed by Du Hamel; see his Papers on the Reproduction of Bone, in Mém. de l'Acad. des Sciences, 1739-43.

sional appearance of vessels even in their substance when diseased—as in the case described by Mr. Mayo, (Med.-Chir. Trans., vol. xix. p. 63,)—the occasional existence of a vascular false membrane passing from the synovial surface of one cartilage to that of another, without any communication with the walls of the joint, and without any apparent vascularity of the adjacent synovial surface,—the peculiarities which it presents in some other diseases,—the weakness of all the objections invented against its vascularity, and all the evident improbabilities of a non-vascular substance existing in such circumstances and presenting such phenomena as cartilage does,—by all these the vascularity and independent power of this tissue may be deemed sufficiently well established to be admitted in the explanation of the various forms of ulceration to which it is subject.

Of all that have been offered, the most concise and correct view of the ulcerations of cartilage is, in our opinion, that which Mr. Mayo has given in the paper just referred to; it is that which with very slight modification we should exactly adopt. There are three kinds of ulceration of articular cartilage: one in which it commences from the synovial surface; a second in which it commences at the surface next the bone—in both these the substance of the cartilage that remains is white, firm, and scarcely altered in appearance; in the third kind the remaining cartilage is softened, and converted into a fibrous or brush-like substance.

In the first class of cases, which may be called simple superficial ulceration, a portion of the surface of the cartilage looks as if it had been irregularly chipped or chiselled out; the surface thus exposed retains its natural colour, its firmness, and its polish. When a joint thus affected is opened it is scarcely imaginable that so little should be left by such formidable symptoms as those by which the disease was accompanied; for sometimes the redness of the synovial inflammation by which this form is accompanied has passed away, and nothing is found but the uneven cartilage. The most common cause of, or at least coincidence with, this kind of ulceration, is intense or long-continued inflammation of the synovial membrane, such as occurs in penetrating wounds of the joints, or in perforation from ulceration, or in the diffuse suppurations met with in phlebitis,* or such as are sometimes produced by any of its common causes, as cold, &c. The effusion of pus into the joint is of course the result of the synovitis and is generally coincident with the ulceration of cartilage; but neither it nor the effusion of lymph are essential to this affection. Most commonly there is also coincident ulceration of the cartilage on the surface next the bone; but in some cases this is absent, and especially, we think, in those which accompany the general and apparently metastatic suppurations in phlebitis; in these the cartilage always retains its firm connexion to the bone, and can no more be torn from it than it can in health. When the ulceration in this form has extended down to the bone it exposes a healthy surface or one only increased in vascularity; and at any period one may determine that the ulceration commenced on the synovial aspect by observing that the exposed portion of bone is smaller than the area of the synovial aspect of the ulcer, so that the borders of the ulcer shelve downwards and inwards and have their thin edges more or less adherent to the bone that is exposed.

In the second kind of ulceration of articular cartilage, which may be called simple deep ulceration, the cartilage is at first removed only on the surface next the bone; it is undermined, and may be torn up from the osseous surface, or is even found hanging from it in pieces or lying loose in detached portions in the interior of the joint. Its under surface is rough and irregular, as if coarsely worm-eaten; often its whole thickness is perforated, and on opening the joint the cartilage looks riddled with little vascular masses of granulation projecting through its apertures from the bone beneath it.

The exposed surface of bone is highly vascular, and wherever the cartilage is separated is covered by prominent, soft, and very red granulations, which fit into the cavities on the under surface of the cartilage. It is probably to the pressure of the cartilage on these granulations that the exquisite pain which accompanies this form of the disease may be ascribed. The cartilage itself can scarcely be the source of pain of the character here felt; but the peculiar acuteness of the suffering produced by even lightly touching the granulations from bone is sufficiently well known.

In some cases the bone is evidently softened and atrophied or ulcerating; but in others it retains all its firmness and, except for an increase in its vascularity, might be deemed healthy. The latter is rarely found in diseased hip-joints; but more commonly in the most acute cases in which ulceration of the cartilages occurs in coincidence with intense synovitis; in chronic cases the disease commencing with a moderately acute inflammation of the synovial membrane, goes on to affect in turn all the tissues belonging and adjacent to the joint, or it commences in the cartilage, or simultaneously in the bone and cartilage. The ordinary cases of chronic diseased knee-joint are examples of the first kind; those of morbus coxæ, the scrofulous diseases of the hip-joint of young subjects, are instances of the second.

In all these forms of disease, however, and whatever be their origin, their ultimate effects if unchecked are disorder and functional destruction of every tissue. The effects of the first stages can be learnt only by the examination of extremely acute cases, and of those in which accidental death has prematurely placed them at our disposal, or by the careful comparison of a great number of advanced cases. From both the result is that the absorption of cartilage may be the initiative; but that in the majority of cases it is subsequent to inflammation of the synovial membrane.

The third form of ulceration of the cartilages in which its tissue is converted into a fibrous or brush-like substance is by far the rarest. It is a chronic disease, and appears to commence on the synovial surface, for the part of the cartilage next to the bone and the bone itself are usually healthy.

We had intended to consider the pathological changes observed in some other forms of diseases of the joints, and to point out the general deductions which the present state of knowledge permits to be drawn from them; but this article has already run its full length, and we must for the present leave the subject.

